# Reading speed, visual deficits, and cerebral white matter integrity in veterans with and without mild traumatic brain injury

**DOI:** 10.3389/fnins.2026.1828388

**Published:** 2026-05-22

**Authors:** Kori E. Skrypek, Philip C. Burton, Nicholas D. Davenport, Scott R. Sponheim, Cheryl A. Olman

**Affiliations:** 1 Department of Rehabilitation, Children’s Hospitals and Clinics of Minnesota, Roseville, MN, United States; 2 Department of Psychology, University of Minnesota, Minneapolis, MN, United States; 3 VA Medical Center, Minneapolis, MN, United States; 4 Department of Psychiatry, University of Minnesota, Minneapolis, MN, United States

**Keywords:** combat veterans, diffusion-weighted imaging, mTBI, reading speed, visual stress

## Abstract

**Introduction:**

Since 2001, approximately 17.3% of enlisted personnel have experienced a traumatic brain injury (TBI) according to the United States military. Visual deficits (e.g., convergence insufficiency or pursuit abnormalities) are reported as chronic, persistent symptoms of TBI, which can impact daily activities such as reading, computer work, and driving.

**Methods:**

In the present study, diffusion-weighted imaging (DWI) data and behavioral and survey data related to visual function were analyzed for 63 combat veterans with and without mild TBI (mTBI). We also tested the hypothesis that white matter damage, measured as either decreased fractional anisotropy of white matter or “potholes” evident in the DWI data, would predict visual behaviors (reading speed, smooth pursuit catch-up saccades, and/or convergence insufficiency).

**Results:**

Our key finding is that scores on the Convergence Insufficiency Symptom Survey (CISS) predicted whether the use of a color overlay would increase reading speed for participants with mTBI, but not for control participants. General linear model analyses found a relationship between smooth pursuit catch-up saccades and the cumulative number of white matter “potholes” found in white matter across the cerebrum. However, the sample size was too small to conclude that these correlations were uniquely related to TBI status.

**Discussion:**

These findings point toward the importance of additional research to determine exactly how mTBI is associated with reduced reading speed and why altering the color of the page improves performance for individuals with mTBI and convergence insufficiency.

## Introduction

During the first two decades of the 21st century, over 350,000 incident diagnoses of traumatic brain injury (TBI), about 17.3% of enlisted personnel, have been reported by the United States military (Lindquist et al., 2017). These injuries can result from blunt force trauma to the head as well as from exposure to a blast wave from an explosive, causing the brain to accelerate and make impact with the skull. Despite these varying mechanisms of injury, it has been found that blast-related and non-blast related samples are largely indistinguishable (Mac Donald et al., 2014; Taber et al., 2006). Health problems and other symptoms associated with TBI include somatic, cognitive, and affective disruptions (Prince and Bruhns, 2017). Among the somatic symptoms of TBI are vision problems, including light sensitivity, saccadic dysfunction, convergence insufficiency, strabismus, pursuit abnormalities, fixation defects, and visual field deficits (Gilmore et al., 2016; Goodrich et al., 2013).

Convergence insufficiency (CI: difficulty aligning the eyes on a target at close range), and pursuit abnormalities, such as deficits in the visual tracking of a moving object, were the target of the present study. Vergence and smooth pursuit information are carried in parallel neural circuits that utilize distinct portions of the same oculomotor association areas such as the frontal eye fields (Büttner-Ennever and Horn, 1997). Although these movements make distinct contributions to tasks, coordination of these movements is required in naturalistic settings, implying a certain level of overlap. When considering the complexities introduced as multiple oculomotor movements are employed, a deficit in one movement may explain variance in another.

Chronic convergence insufficiency is generally associated with traumatic brain injury. Some evidence has linked these deficits to axonal damage in subcortical regions such as the mesencephalic reticular formation, oculomotor nucleus, and abducens nucleus while indicating that more research examining cortical regions is needed (Fortenbaugh et al., 2021; Searle and Rowe, 2016). Additionally, cranial nerve dysfunction has been cited as a common result of mTBI, impacting oculomotor function (Coello et al., 2010; Keane and Baloh, 1992). Convergence insufficiency rarely occurred alone in these cases. A study of CI associated with TBI found that only 9% of their sample of 557 in-patient TBI records was diagnosed with CI without a concurrent dysfunction related to smooth pursuit movements, saccadic movements, cranial nerve palsy, spatial inattention, visual field deficit, vestibular deficits, and/or nystagmus (Alvarez et al., 2012). Possibly related to these visual deficits, Goodrich et al. (2013) found that 50% of their patients with both blast and non-blast related TBIs reported reading difficulty (Goodrich et al., 2013).

Oculomotor deficits, such as in smooth pursuit, are another cited symptom of traumatic brain injuries (Goodrich et al., 2013; Grossberg et al., 2012). Previous studies have found a moderate effect on oculomotor function in patients with traumatic brain injury (Mani et al., 2018), and intra-parietal lobule—associated with spatial reasoning and allocation of spatial attention—is often a locus of damage following concussion (Danielli et al., 2023). When considering this relationship, eye movements have the potential to aid TBI diagnosis because they require intact attentional, cognitive, and motor functions (Stuart et al., 2020). One challenge to drawing strong conclusions about the effect of TBI on oculomotor function, however, is that there is a fair amount of heterogeneity in the literature due to participant characteristics and varying methodologies. Thus far, only participants with severe levels of TBI differ enough from healthy controls in smooth pursuit performance to formulate a diagnosis (Hunfalvay et al., 2020), but milder forms of TBI may nonetheless perturb eye movements. It appears that eye movement deficits persist long after initial injury, indicating that more research about cortical damage to oculomotor neural circuitry is needed.

The present study was initiated to test the hypothesis that TBI impacts eye movement planning and execution via cortical axonal damage. Traumatic brain injuries often produce diffuse axonal injuries (Davenport et al., 2012; Sponheim et al., 2011). Diffusion tensor imaging (DTI) can reveal decreases in white matter integrity in patients with TBI in cases when other structural MRI modalities and CT scans do not reveal abnormalities (Mac Donald et al., 2011). To execute eye movements, the visual system depends on a multitude of tracts connecting many regions of the brain, including primary visual cortex, frontal eye fields, and oculomotor nuclei. This network is involved in tasks such as the initiation of movement, attention allocation, maintenance of the motion, and adjustments as needed. This must be done quite rapidly to promote effective responses, which requires intact myelin and white matter (Rokem et al., 2017). The intraparietal sulcus, middle temporal (MT), medial superior temporal (MST), and lateral intraparietal areas (LIP) are theorized to initiate, maintain, and coordinate oculomotor movements (Fukushima et al., 2013; Grossberg et al., 2012; Krauzlis, 2004; Srihasam et al., 2009). In caudal middle frontal cortex, the frontal eye fields (FEF), also called the frontal pursuit area (FPA), and adjacent supplementary eye fields (SEF) are thought to allocate spatial attention and plan future eye movements (Fukushima et al., 2013; Grossberg et al., 2012; Jerde et al., 2012; Krauzlis, 2004). An efference copy loop with both predictive and reactive functions is believed to allow pursuit to be maintained and adjusted (Grossberg et al., 2012).

We examined the fractional anisotropy (FA) values for regions specifically related to visual function as well as whole-brain measures to characterize white matter integrity more broadly. Our study considered four wide areas of interest in the left and right caudal middle frontal cortex (CMF) and the left and right superior parietal cortex (SP). To get a full picture of white matter integrity, we also utilized an analysis technique that looks for clusters of white matter voxels in which fractional anisotropy was more than two standard deviations below the voxel-based mean, dubbed “potholes” (White et al., 2009).

Because reading is a demanding visual behavior that relies on eye movements, a second hypothesis tested by the study was that tinted plastic color overlays could positively impact reading speed.. Reports have indicated that reading difficulties can be mitigated by applying a transparent, tinted plastic sheet to the page, altering the relationship between black ink and a white background that is common in text (Evans and Allen, 2016). Studies that do report reading speed improvement with tinted filters commonly find that slower readers benefit more from these filters than subjects without reading difficulties (Scott et al., 2002). For example, a study on readers with dyslexia reported that tinted filters improved “visual stress,” which is defined as “the inability to see comfortably and without distortion,” compared to controls who did not report this symptom (Uccula et al., 2014). Initial hypotheses about the neural mechanisms underlying this observation include cortical hypersensitivity (i.e., patterns commonly seen in text evoke unusually strong responses in some observers), and magnocellular dysfunction (i.e., visual traces from previous images mask current images). A functional magnetic resonance imaging study has, for example, demonstrated hyperstimulation in the cortex of participants with visual stress syndrome (Chouinard et al., 2012; Uccula et al., 2014). Results have been mixed, however, and the efficacy of tinted filters is somewhat controversial (Griffiths et al., 2016). This secondary effort, which turned out to be the most productive part of the investigation, was to assess whether reducing visual stress with tinted filters could aid TBI patients with reading difficulties.

## Materials and methods

### Participants

The participants recruited for this study were a subset of participants in two other projects more broadly investigating veterans with TBI due to blast exposure [“SATURN” (Davenport et al., 2012); “DEFEND” (Davenport et al., 2015)]. All participants were veterans of the United States military who had previously been deployed to combat zones in Operation New Dawn, Operation Enduring Freedom, or Operation Iraqi Freedom. Some key exclusionary criteria for these studies included unstable medical conditions which could be reasonably expected to affect their brain functioning, a diagnosed psychiatric condition before deployment, substance dependence, and contraindications to magnetic resonance imaging (e.g., claustrophobia, pacemaker implant, shrapnel). Three of the recruited participants were female; 61 were male. Biological sex was not included as a variable in analysis because of the disproportionate representation of males due to lack of representation of females in the veteran population.

In the current study, additional exclusion criteria included a post-traumatic stress disorder (PTSD) diagnosis and lack of normal or corrected-to-normal vision. Participants ranged in age from 27 to 65 (*M* = 40.33, SD = 9.47). Participants were categorized as control participants (*N* = 20) or members of the TBI group (*N* = 44 during recruitment; *N* = 43 for analysis) based on self-report data and consensus review of structured interviews, which included the Structured Clinical Interview for DSM-IV-TR (SCID; First et al., 2002), CAPS, and the Minnesota Blast Exposure Screening Tool (MN-BEST; Nelson et al., 2011). Symptoms of mTBI were assessed using the MN-BEST and included altered consciousness (e.g., confusion and disorientation), loss of consciousness (LOC) less than 30 min, post-traumatic amnesia (PTA) up to 24 h, and neurological symptoms (e.g., headache, tinnitus, nausea, sensitivity to light or noise) immediately after the event. The three most significant potential blast-related and impact-related TBI events were considered, each of which received a severity score ranging from 0 (no concussion) to a potential maximum of 30 (severe TBI). One score was higher than 4 (LOC 5–30 min or PTA < 12 h) in the current sample; this individual was excluded from analysis to include only TBI cases that were considered mild. All TBI ratings were completed by doctoral-level neuropsychologists based on descriptions of events secured by trained study interviewers. The abbreviation mTBI used throughout this manuscript indicates “mild TBI”. All study procedures were approved by the University of Minnesota’s Institutional Review Board, and participants gave written informed consent prior to participating in the study, in accordance with the Declaration of Helsinki.

### Behavioral measures

Each experimental session began with participants completing surveys to collect demographic information (date and location of birth, gender assigned at birth, racial and ethnic identity) and to assess related vision and reading abilities. The Edinburgh Handedness Inventory (EHI) (Oldfield, 1971) was administered to account for potential lateralization of brain function in the neuroimaging data analysis. Next, participants filled out a survey assessing personal and family history of strabismus (Personal and Family History of Strabismus), and the Visual Functioning Questionnaire (VFQ-25), which details how visual deficits impact participants’ daily life. Both of these measures were obtained from the web-based PhenX Toolkit (Hamilton et al., 2011). Finally, participants completed the Convergence Insufficiency Symptom Survey (CISS), which has been shown to distinguish adults with normal binocular vision from those with convergence insufficiency with good sensitivity (97.8%) and specificity (87%) (Rouse et al., 2004). Responses are given on a Likert scale ranging from “never” = 0 to “always” = 4 (Scheiman, 2008). Questions ask about key symptoms such as headache, blurred vision, poor concentration, etc.

Visual acuity was tested either by the ETDRS chart or MNRead acuity test. The first 32 participants were tested with the ETDRS chart at two meters (Precision Vision, 2023). The MNRead program was implemented on an iPad for 17 of the remaining 32 participants (Calabrèse et al., 2016). The MNRead test is a preferred measure of acuity because it provides a more meaningful measure in a naturalistic setting, but acquisition of the equipment was delayed for this study, resulting in 13 missing data points. During analysis, we also encountered several recorded scores unlikely to be true (e.g., one participant scored a LogMAR = 0.6 despite completing a reading task with a 12-point font from 50 centimeters away with ease), and these were excluded from analysis (see Table 1). Because acuity data were unusable for a total of 16 of the 63 participants, regression analyses were done without acuity as a nuisance regressor because of the limitation on sample size. However, key regression analyses were repeated with acuity as a regressor, and if results differed qualitatively, those differences are reported.

**TABLE 1:** Pearson’s correlations between baseline reading speeds without (no color overlay) and CISS and the four factors surveyed in the Visual Function Questionnaire (VFQ-25).

VFQ factor	Reading speed without color	CISS
Near activities	*r*(26) = −0.188, *p* = 0.338	*r*(30) = 0.702, *p* < 0.001
Distance activities and mobility	*r*(26) = −0.566, *p* = 0.002	*r*(30) = 0.407, *p* = 0.021
Pain and discomfort	*r*(26) = 0.007, *p* > 0.10	*r*(30) = −0.316, *p* = 0.078
Mental health and dependency	*r*(24) = 0.090, *p* > 0.10	*r*(28) = −0.493, *p* = 0.006

*Denotes significant after correction for multiple comparisons.

Reading speed was measured with and without color overlays. Before performing the Wilkins Rate of Reading Test (WRRT) (Wilkins et al., 1996) asked to choose a preferred color overlay: “select whatever color you want, based on whether it makes text easier to see or if you just like the color.” Participants then read two of the four versions of the WRRT aloud while being timed, one with the color overlay and one without (order was randomized). Fifty-nine participants completed the baseline WRRT, and of those, 55 completed the test with color overlay. In each case, the words per minute were recorded for each reading, and the overlay advantage was calculated as the ratio of reading speed with the color overlay to reading speed without color.

Smooth pursuit eye movements were measured using an EyeLink 1000 Plus (SR Research Ltd.)^1^ eye tracker that followed both eyes while participants tracked a 3 mm diameter (0.34° degree visual subtense) blue dot across a series of paths on a computer monitor from a viewing distance of 50 cm. (The relatively small viewing distance was necessitated by space constraints in the room where the measurements were performed.) The 6 paths consisted of a diagonal line moving from lower left to upper right, a diagonal line from upper left to lower right, a vertical line, a horizontal line, and an ellipse (separate trials measured clockwise and counterclockwise motion). Each of the 6 trials lasted 6 s (the dot followed an out-and-back path for the straight lines), taking 0.5 s at the beginning and end of each path to ramp up to a peak velocity of 5 °/s. An occluder was placed over a portion of the upper right quadrant of the screen to enable measurement of predictive object tracking; however, use of the occluder was inconsistent between study visits (it was accidentally omitted for some visits, without being noted). Therefore, all data collected in the upper right quadrant of the screen were excluded from analysis. Smooth pursuit accuracy is defined by the number of catch-up saccades or “jumps” used to track the blue dot, with more saccades indicating less accuracy. Data could not be collected for 10 of 64 participants due to occasional equipment failure or lack of time. Jumps were quantified two ways: by manual inspection, and with an algorithm that detected abrupt changes in eye position. Preliminary analysis determined that the metrics were strongly correlated; final analyses used the manual jump counts.

To assess participants’ overall cognition, a cognitive battery was administered including (1) the Wechsler Test of Adult Reading (WTAR; Weschler, 2001), generally considered a measure of premorbid intelligence, (2) the Wechsler Adult Intelligence Scale - 3rd Edition (WAIS-III, Weschler, 1997), a measure of current intelligence, (3) the California Verbal Learning Test - 2nd Edition (CVLT-II, Delis et al., 2000), a measure of verbal learning and memory, and (4) the Trail Making Test A and B [(Reitan and Wolfson, 1993), using the (Heaton et al., 2004) normative comparison sample], which measures of processing speed, attention, general executive function, and set shifting. In total, 56 participants were included in subsequent cognitive analyses after removing those with more than one failed performance validity measure. From the WTAR data, an estimate of each participant’s premorbid IQ (WTAR FSIQ) was derived. A confirmatory factor analysis of five cognitive measures (WAIS-III Digit Span and Coding scaled scores, Trail Making Test A and B T-scores for completion times, and CVLT-II list 1–5 recall z score) yielded a latent Cognitive Performance factor value for each participant (Nelson et al., 2020), referred to in the Results as “Cognitive Factor.”

### Interim analysis of visual function questionnaire

When the study began recruiting from former SATURN participants (the second half of the study), we needed to shorten the protocol to decrease burden on participants. We performed an interim analysis on behavioral data and found that answers on the 25-question VFQ were highly correlated with answers on the much shorter CISS. Thus, we did not collect VFQ data after the midpoint of the study, and results for the first 32 participants are reported here for the sake of completeness.

A factor analysis (Langelaan et al., 2007) has previously revealed that the VFQ-25 items measured four underlying constructs: Near Activities, Distance Activities and Mobility, Mental Health and Dependency, and Pain and Discomfort. Computing these 4 factor scores for our participants, we found significant correlations between three of these factors (Near Activities, Distance Activities and Mobility, and Mental Health and Dependency) and baseline reading speed (i.e., without a color overlay), CISS, or both (Table 1). For both the Near Activities and the Distance Activities and Mobility factors, higher scores were associated with slower reading speeds. Both of these factors were also positively correlated with convergence insufficiency scores as measured by the CISS, whereas the Mental Health and Dependency factor was negatively correlated with CISS. We did not find a relationship between the Pain and Discomfort factor and either baseline reading speed or CISS.

### Diffusion-weighted imaging

All scans were conducted with a Siemens Trio 3T scanner (Siemens, Erlangen, Germany) at the Center for Magnetic Resonance Research at the University of Minnesota. Two protocols were used to collect diffusion-weighted images, depending on the project the participant was recruited from. Both studies acquired oblique axial images in 30 directions with an isotropic 2 mm voxel size. However, the studies differed in head coil (12-channel birdcage for the 32 participants recruited from SATURN vs. 32-channel receive-only for the 32 participants recruited from DEFEND), number of slices (72 in SATURN vs. 66 in DEFEND), and b-value (800 in SATURN vs. 1,500 s/mm^2^ in DEFEND). SATURN data are fully described in Davenport et al. (2012) and DEFEND data are fully described in Sun et al. (2020). A categorical variable indicating source of DTI data was included in regression analysis to account for source differences. To address the fact that DEFEND data were acquired with parameters that should produce more precise estimates of WM integrity, analyses were repeated in just the half of the sample recruited from the DEFEND study and those results are reported alongside the full sample analysis.

Fractional anisotropy (FA) and mean diffusivity were estimated using FSL Diffusion Toolbox (Behrens et al., 2003), as reported in Davenport et al. (2015). Four *a priori* regions of interest were defined, using the Destrieux atlas from Freesurfer: left/right superior parietal cortex and left/right caudal middle frontal cortex. These regions were chosen because they are adjacent to the frontal eye fields and intraparietal sulcus, which are implicated in allocation of spatial attention and planning of eye movements (Jerde et al., 2012). For the whole brain analysis, the number of “potholes” in each participant was calculated by first generating the voxel-by-voxel mean and standard deviation (SD) from the FA values across all participants (White et al., 2009). While only controls were used to establish the baseline in White et al. (2009), we used all participants to compute the baseline following more recent best practices established in clinical studies (Watts et al., 2014). A “pothole” was defined as a cluster of at least 50 contiguous voxels in which the FA was at least two standard deviations below the voxel mean.

### Summary of data availability and statistical tests

Limitations impacting the available data include: the study being completed on a relatively small budget, significant staff turnover, and time constraints during study visits. Thus, analyses needed to account for many missing datapoints. Here, we tabulate the available data on all primary measures (Table 2) as well as nuisance regressors used in statistical analyses (Table 3).

**TABLE 2:** Available primary measures.

Measure	DEFEND participants	SATURN participants	Total datasets
Convergence Insufficiency Symptom Survey (CISS)	32 (11, 21)	31 (9, 22)	63 (20, 43)
Visual Function Questionnaire (VFQ)	32 (11, 21)	0^1^	32 (11, 21)
Wilkins rate of reading test	No overlay: 28 (7, 21) With overlay: 24 (7, 17)	No overlay: 30 (9, 21) With overlay: 30 (9, 21)	58 (16, 42) 54 (16, 38)
DTI data	30 (10, 20)	28 (9, 20)	59 (19, 40)

Numbers in parentheses: (control, mTBI). Because only 3 females participated in the study, sex was not considered as a biological variable.

^1^Analysis at the midway point in the study indicated that VFQ metrics were strongly correlated with CISS, so the measure was dropped from the protocol to reduce study visit duration.

**TABLE 3:** Available Secondary measures.

Measure	DEFEND N_*total*_ (N_*ctrl*_, N_*mTBI*_)	SATURN N_*total*_ (N_*ctrl*_, N_*mTBI*_)	Group (N): mean (stdev) *t*-test results
Personal and Family History Strabismus	32 (11, 21)	31 (9, 22)	qualitative data
Age	32 (11, 21)	31 (9, 22)	Ctrl (20): 42.4 (9.73) mTBI (43): 39.6 (9.60) *t*(61) = 1.05, *p* = 0.30
Acuity^1^	EDTRS: 32 (11, 21) MN-Read: 0	EDTRS: 0 MN-Read: 15 (6, 9)	Ctrl (17): −0.01 (0.12) mTBI (30): 0.03 (0.17) *t*(45) = −0.72, *p* = 0.48
Smooth pursuit stability (jumps)	31 (11, 20)	22 (8, 14)	Ctrl (19): 26.05 (13.81) mTBI (34): 25.50 (12.83) *t*(51) = 0.15, *p* = 0.88
EHI (handedness)	25 (9, 16)	25 (8, 17)	Ctrl (17): 0.62 (0.25) mTBI (33): 0.59 (0.58) *t*(48) = 0.19, *p* = 0.85
WTARFSIQ	30 (11, 20)	25 (7, 18)	Ctrl (18): 103 (10.5) mTBI (28): 102 (8.8) *t*(54) = 0.28, *p* = 0.78
Composite Cognitive Performance Factor	30 (10, 20)	25 (7, 18)	Ctrl (17): 0.09 (0.19) mTBI (38): 0.04 (0.31) *t*(53) = 0.70, *p* = 0.49

These measures were potential confounding variables that were used as nuisance regressors in linear regression models. In all cases, Levene’s test (as implemented by leveneTest() in the R *car* package) failed to reject the null hypothesis of equal variance, so Student’s *t*-test was used to assess group differences.

^1^MN-Read acuity data availability reflects the fact that we discarded three estimated acuity scores > 0.5, on the presumption that data were recorded incorrectly, because reading speed scores in those individuals were not consistent with such poor recorded acuity.

While there were no group differences in any of these potentially confounding variables, we also tested for potential correlations between these measures and our two primary behavioral outcome measures of interest: CISS and unaided Reading Speed. Age, acuity, jumps, and handedness showed no association with either CISS or reading speed (| *r*| ≤ 0.13; *p* ≥ 0.37). Both WTARFSIQ and the Composite Cognitive Factor showed strong association with Reading Speed [WTARFSIQ: *r* = 0.39, *t*(50) = 2.95, *p* = 0.005; Cognitive Factor: *r* = 0.45, *t*(49) = 3.49, *p* = 0.001] and moderate associations with CISS [WTARFSIQ: *r* = −0.28, *t*(54) = −2.16, *p* = 0.036; Cognitive Factor: *r* = −0.22, *t*(53) = −1.66, *p* = 0.103]. Choosing the Cognitive Factor over WTARFSIQ because it was composed from a broader set of measures, and had the stronger correlation with Reading Speed, we used the Cognitive Factor as a nuisance regressor in all linear models studying the relationship between CISS and Reading Speed or Overlay Advantage.

To control for multiple comparisons, we outlined in advance 8 planned comparisons, tabulated below (Table 4). P-values from these 8 analyses were corrected for multiple comparisons using the Holm correction (p.adjust() function in the R statistical package). Follow-up analyses were performed only for the one significant primary result, as tabulated below.

**TABLE 4:** Planned analyses.

Analysis	N or degrees of freedom	p, unc	p, cor	Additional analyses
Group differences in behavioral measures (t-test)				
CISS	Ctrl(20): μ = 15.60, σ = 12.14 mTBI(43): μ = 22.77, σ = 11.86 *t*(61) = −2.22	0.030	0.213	
Reading speed (unaided)	Ctrl(16): μ = 137.64, σ = 34.55 mTBI(42): μ = 129.77, σ = 29.47 *t*(56) = 0.866	0.390	–	
**Overlay advantage (OA)**	**Ctrl(16): μ = 1.02, σ = 0.06 mTBI(38): μ = 1.11, σ = 0.13 *t*(52.0) = -3.28**	**0.002**	**0.017**	
Relationship between behavioral measures (correlation)				
CISS ∼ Reading speed (unaided)	*r* = −0.30, *t*(56) = −2.33	0.024	0.190	
**CISS ∼ Overlay Advantage**	***r* = 0.47, *t*(52) = 3.79**	**<0.001**	**0.004**	OA ∼ CISS*Group + cog OA_*group*_ ∼ CISS+jumps+age+cog
Relationship between WM integrity and convergence insufficiency				
CISS vs. global WM integrity, GLM	*F*(1, 39) = 1.04	0.31	–	GLM structure: potholes ∼ CISS*Group + age + handedness + Study
CISS vs. FA in 4 ROIs, GLM	lCMF: *F*(1, 39) = 0.039 rCMF: *F*(1, 39) = 0.033 lSP: *F*(1, 39) = 2.25 rSP: *F*(1, 39) = 0.021	0.84 0.86 0.14 0.89	– – 0.85 –	GLM structure: FA ∼ CISS*Group + age + handedness + Study

Numbers in parentheses indicate numbers available for each analysis. *P*-value correction for planned analyses was performed using the Holm method (R package method p.adjustIO). – indicates corrected *p*-value > 1. In descriptions of GLMs, capitalization indicates variables treated as factors. Bold values indicate statistical significance after correction for multiple comparison.

## Results

**FIGURE 1:**
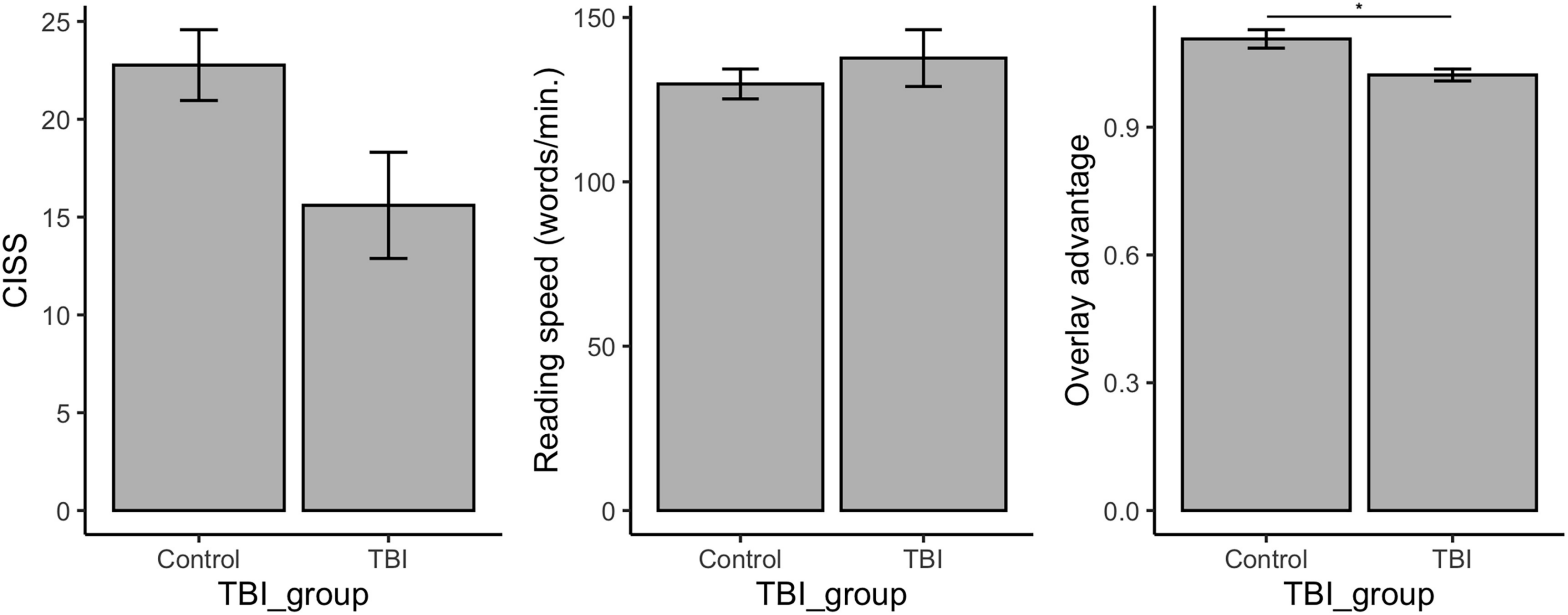
Comparison of group scores on CISS, reading speed, and overlay advantage. In general, a total score of 21 or higher on the CISS propounds convergence insufficiency in adults. Reading speed was measured with the Wilkins Rate of Reading Test. Data shown in the middle panel represent the number of words read in 1 min *without* a transparent, colored plastic overlay. Overlay Advantage was computed as Reading Speed With Overlay divided by Reading Speed Without Overlay, thus, 1 indicates no change in reading speed and numbers greater than 1 indicate an increase in reading speed with colored overlay. Error bars indicate standard error from the mean. *CISS*: ctrl *N* = 20, μ = 15.60, σ = 12.14; mTBI *N* = 43, μ = 22.77, σ = 11.86; Student’s *t*(61) = −2.217, *p* = 0.030, Cohen’s *d* = −0.600. *Reading speed*: ctrl *N* = 16, μ = 137.64, σ = 34.55; mTBI N = 42, μ = 129.77, σ = 29.47; Student’s *t*(56) = 0.866, *p* = 0.390. *Overlay advantage*: ctrl *N* = 16, μ = 1.02, σ = 0.06; mTBI N = 38, μ = 1.11, σ = 0.13; Welch’s *t*(52.0) = −3.279, *p* = 0.002*, Hedges’ *g* = −0.716. *Indicates significance after correction for multiple comparisons.

We first tested for group differences in our three key behavioral measurements—Convergence Insufficiency Symptom Survey (CISS), reading speed (without color overlay), and Overlay Advantage (the relative increase in reading speed with a transparent, colored plastic overlay selected by the participant). Veterans without documented mTBI (control participants) had, on average, lower (better) scores on the CISS than participants with documented mTBI, although the difference was not statistically significant after correction for multiple comparisons (see Figure 1 legend for statistics). A group difference in Overlay Advantage did, however, remain significant after correction for multiple comparisons: members of the mTBI group were more likely to demonstrate an increase in reading speed when using a transparent colored overlay.

**FIGURE 2:**
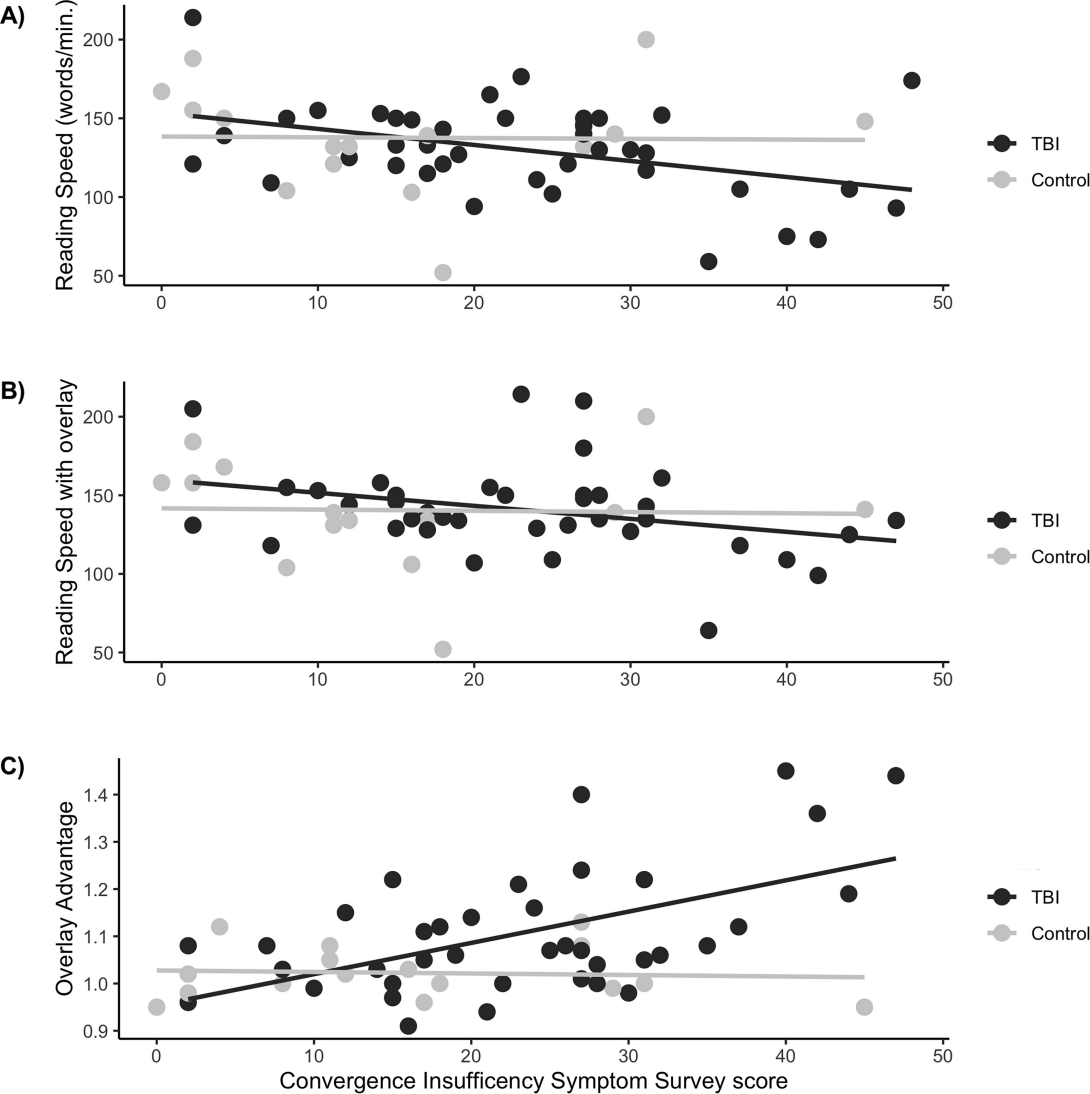
Reading speed, color overlay and CISS scores. **(A)** The relationship between CISS and reading speed (words per minute) is shown for mTBI participants in black dots and lines; data for controls are shown in gray dots and lines. CISS was negatively related to reading speed for participants with mTBI (β = −1.02, *t*_40_ = −2.76, p_*unc*_ = 0.009) but not for control participants (β = −0.048, *t*_14_ = −0.067, *p* = 0.95). **(B)** The difference between groups with respect to the strength of the relationship between CISS and reading speed less pronounced when text was read through a color overlay (β = −0.83, *t*_36_ = −2.00, *p* = 0.053 for the mTBI group, and β = −0.078, *t*_14_ = −0.11, *p* = 0.92 for controls). **(C)** Overlay Advantage was computed for each individual as the ratio of reading speed with color overlay to reading speed without color overlay. For participants with TBI, there was a significant relationship between CISS and overlay advantage (β = 0.007, *t*_36_ = 3.92, p_*unc*_ < 0.001). This relationship was not significant for controls (β = 0.0003, *t*_14_ = −0.27, *p* = 0.79). *Note*: the beta weights and statistics reported here are for simple linear regressions computed to match the visualized data, without nuisance regressors, and are less conservative than the linear models reported in the text.

Because CISS tended to be higher in the mTBI group, as did Overlay Advantage, we followed up by investigating associations between CISS and reading speed, as well as the computed Overlay Advantage (Figure 2). While CISS and reading speed were correlated across all participants [*r* = −0.30, *t*(56) = −2.33, *p* = 0.024], this correlation was not significant after correction for multiple comparisons (see Table 4). However, the correlation between Overlay Advantage and CISS was strong [*r* = 0.47, *t*(52) = 3.79, *p* < 0.001] and significant after correction for multiple comparisons.

To determine whether the relationship between CISS and Overlay Advantage differed for the control and mTBI groups, we built a linear regression model for Overlay Advantage testing the predictive power of TBI status, and CISS, as well an interaction term between TBI status and CISS. Cognitive Factor was included as a nuisance regressor; this reduced the number of participants included in the analysis from 63 to 55, because of missing data elements, but was deemed important (1) a strong correlation exists between cognitive measures and reading speed and (2) TBI can impact cognition, so it is important to test for this association. The model (OA ∼ CISS*TBI + Cog) revealed no significant main effects, but a significant interaction between TBI status and CISS in the ability to explain whether a colored overlay improved reading speed [*F*(1, 42) = 6.634, *p* = 0.014, η^2^ = 0.14].

Follow-up linear regression models tested the relationship between Overlay Advantage and CISS scores in the separate groups. An ideal generalized linear model would include all available visual behavioral variables: CISS, age, visual acuity, Composite Cognitive Factor, and stability of smooth pursuit eye movements (“Jumps”; although reading does not involve smooth pursuit, these data provide a potential indicator of the health of eye movement circuits). However, missing data elements (Table 2) made the full model inadvisable for the relatively small control group. Thus, we took two approaches. First, we randomly sampled a subset of 20 mTBI participants to match the size of the control group and conducted a simplified linear model using only the Cognitive Factor as a nuisance regressor (OA ∼ CISS + Cog). In this analysis, Overlay Advantage was significantly predicted by CISS for veterans with documented mTBI [*F*(1, 12) = 20.817, *p* = 0.001, η^2^ = 0.63] but not veterans without documented mTBI [*F*(1, 10) = 0.113, *p* = 0.743]. Then, to rule out confounding variables in the mTBI group, a linear model with all possible confounding variables (OA ∼ CISS + Cog + Age + Jumps + Acuity) was conducted in the mTBI group only. In spite of the reduced degrees of freedom, CISS scores continued to explain a significant proportion of the variance in Overlay Advantage for mTBI participants [mTBI: *F*(1, 15) = 7.365, *p* = 0.016, η^2^ = 0.31].

To summarize the behavioral data from this study, the visual behavioral data collected in this study indicate that (1) the addition of a color overlay (individually selected by the participant as preferred or most comfortable) increased reading speed for some but not all participants, (2) reading speed was more likely to be positively impacted by the color overlay in the mTBI group, and (3) scores on the CISS were predictive of whether the colored overlay would improve reading speed for veterans with documented mTBI.

To examine whether compromised cerebral white matter might account for convergence insufficiency or reading difficulties we analyzed diffusion-weighted MRI data. Fractional anisotropy (FA) values were averaged across voxels for each individual in each of four *a priori* regions of interest: left and right caudal middle frontal cortex and left and right superior parietal cortex. In addition, a whole-brain white matter integrity metric called “potholes” was computed by counting the number of clusters of voxels with FA more than 2 standard deviations below the mean of the entire sampled population, using a cluster threshold of 50 voxels.

In planned analyses, average FA in the 4 *a priori* white matter regions abutting cortical regions that are key for planning eye movements, as well as the whole-brain potholes count, were tested for their ability to explain CISS, using linear regression models with a group interaction term as well as relevant nuisance variables to absorb variance in the imaging data (age, handedness, and study of origin, since the DWI data were acquired with different protocols for different halves of the group). No regression showed any significant terms (see Table 4). Since there was no group interaction, the regression models were repeated without the interaction, using a Type II ANOVA to test for significance of the regression weights. Again, no WM metric explained participants’ scores on the CISS; the strongest observed effect was for the potholes metric [*F*(1, 39) = 2.27, *p* = 0.14, η^2^ = 0.03]. Thus, we conclude that there is no relationship between CISS and white matter integrity in the present dataset.

**FIGURE 3:**
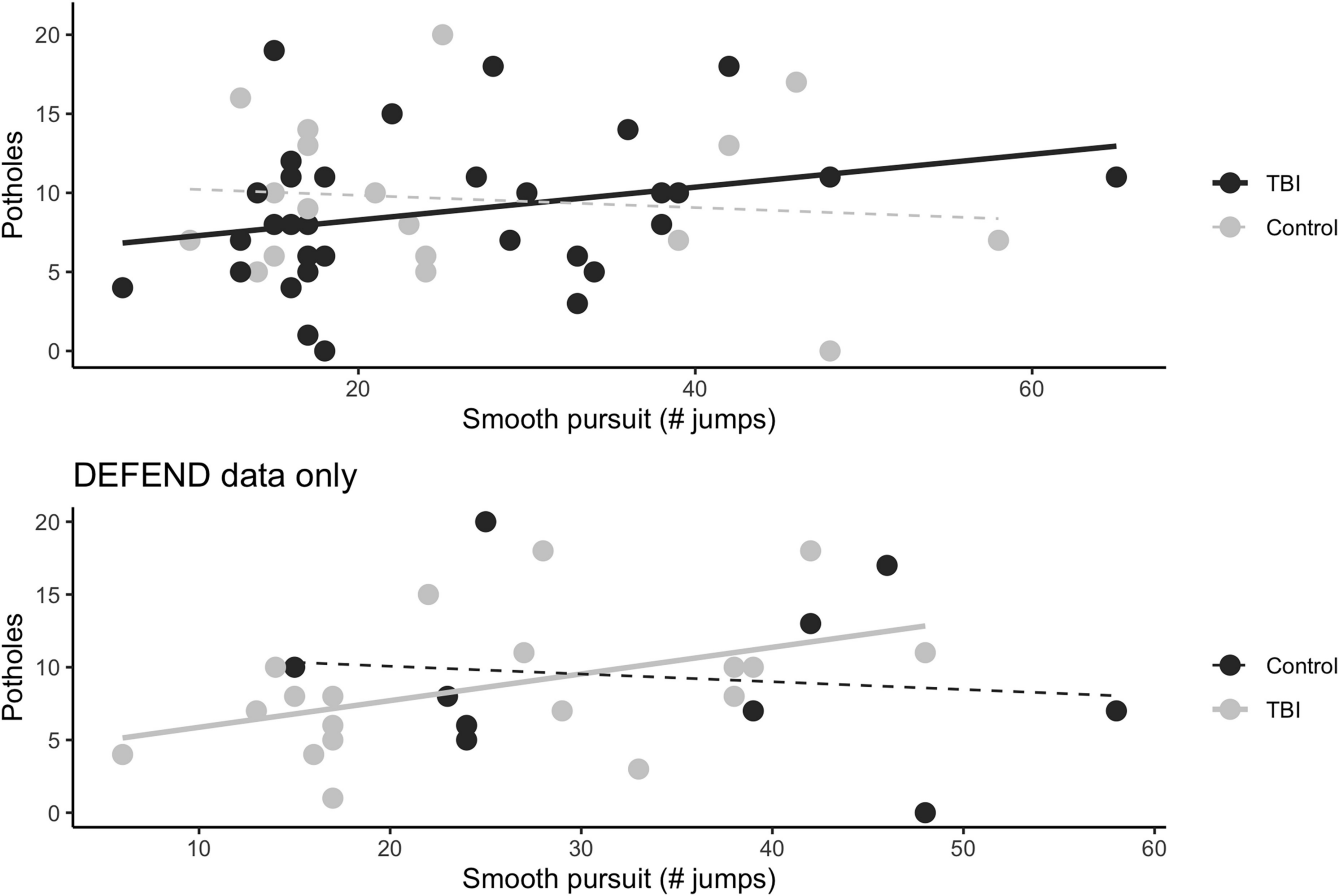
Smooth pursuit catch-up saccade count and whole-brain pothole count. The top plot includes DWI data from both the (older) SATURN and (newer) DEFEND protocols; the bottom plot includes only participants recruited from the DEFEND group. Black dots indicate controls; gray dots indicate participants with mTBI; lines show trends for each group; the dashed black lines indicate that linear models found no associations between potholes and smooth pursuit eye movements, however it is important to note that all analyses shown in this figure are exploratory and the control population was particularly small.

To make good use of the existing dataset, exploratory analyses also tested for associations between stability of smooth pursuit eye movements and potholes, one measure of whole-brain WM integrity. Visually (Figure 3) there appeared to be a positive association between the number of catch-up saccades and the number of potholes, which is consistent with the idea that WM damage could impair eye movements. General linear models that included catch-up saccade count, as well as handedness, age, and original study (the earlier SATURN study vs. the later DEFEND study) as nuisance regressors, estimated a weak association between catch-up saccade count and the whole-brain potholes measure for all participants [*F*(1, 35) = 3.39, *p* = 0.075, η^2^ = 0.09], and a slightly stronger association for the mTBI group [*F*(1, 21) = 6.05, *p* = 0.02, η^2^ = 0.22] but not controls [*F*(1, 11) = 0.14, *p* = 0.72]. (Note that degrees of freedom are significantly reduced due to missing handedness data in 10 of the participants for whom DWI and smooth pursuit were available, and that these results are exploratory and not corrected for multiple comparisons.)

This exploratory finding is consistent with the idea that decreases in white matter integrity (reduced FA or increased pothole count) could be related to a decreased ability to engage in smooth pursuit motions. Since the DWI data in our dataset were drawn from two previous studies, we repeated the analysis in the subset of the data with stronger b-values (data collected in the DEFEND protocol) and found similar associations [*F*(1, 19) = 11.6, *p* = 0.003, η^2^ = 0.46 for all participants; *F*(1, 11) = 18.0, *p* = 0.001, η^2^ = 0.62 for the mTBI group; *F*(1, 4) = 0.971, *p* = 0.38]. This lends credence to a potential relationship between white matter integrity and smooth pursuit eye movements, but the relatively small sample size and further power limitation due to missing data elements means that conclusions cannot be drawn from these data and these are descriptive results only.

## Discussion

Our study confirms previous research showing a clear relationship between convergence insufficiency (quantified as CISS scores) and mTBI (Alvarez et al., 2012; Fortenbaugh et al., 2021; Searle and Rowe, 2016). Vergence eye movements are required for many aspects of daily life such as reading, hobbies, school/work performance, sports, and much more. Notably, reading has a great impact on daily function and is an activity with which many patients with mTBI cite newfound struggles after injury (Goodrich et al., 2013). In fact, many CISS items ask about symptoms that occur while reading.

Our study found that CISS scores are able to predict whether individuals with documented mTBI will benefit from using a transparent color overlay when reading: many mTBI participants who had read slowly in the baseline condition were reading at a more normal speed with assistance of the color overlay. While CISS scores and reading speed could both be affected by executive function deficits, which are expected after TBI, the fact that reading speed increased for individuals with the simple application of a transparent color overlay indicates a sensorimotor rather than cognitive mechanism underlying at least some of the slower reading speeds. Anecdotally, one participant indicated that he would be able to enjoy reading as a pastime again if he could take home the purple overlay he had selected for the test. These findings augment previous arguments that those who struggle the most while reading experience the greatest benefit from the overlay (Scott et al., 2002). Adding to the mixed body of literature, our findings indicate that color overlays could be a possible tool to remediate visual deficits that impact reading abilities in a veteran population who have experienced a traumatic brain injury, although the mechanisms remain unclear and should be a topic of further study. We also note that the population in this study was overwhelmingly male, which may limit generalizability of the findings.

The relationship between CISS and overlay advantage is enhanced by above-median overlay advantage scores for the participants with the highest CISS scores: 8 of the 9 highest scores belonged to participants with mTBI. Of those 8, 1 reported (on the Personal and Family History of Strabismus) a past eye injury and on-going double vision, 1 reported a history of strabismus and on-going double vision, 3 reported double-vision, 1 reported tilting their head to view things, and 2 reported no vision abnormalities. While quantitative assessment of these heterogeneous reports in our relatively small sample size was not feasible, this distribution of vision abnormalities was similar to the distribution across the whole population: 4 (6%) reported eye injuries or abnormalities like misformed lens, 23 (36%) reported tilting their heads or experiencing double vision or both, so there was no clear association between reports of abnormal vision and high CISS scores. Similarly, there were no systematic differences in clinical interviews related to TBI diagnosis and CISS scores.

Previous work theorizes that the presence of a color overlay changes the perceptual relationship between the text and background, in turn reducing visual stress with various neurological mechanisms proposed (Chouinard et al., 2012; Griffiths et al., 2016; Uccula et al., 2014). Notably, the demonstrated benefit of an individually selected color overlay alludes to a sensorimotor concern in nature, especially when considering models of sensory processing commonly used in clinical practice. Dunn’s Model of Sensory Processing explains how each individual interprets sensory input differently with a unique interaction of neurological thresholds and behavioral responses, emphasizing discomfort as a result of sensory overwhelm (Brown et al., 2001; Dunn, 1997). From a neurocognitive perspective, it can be hypothesized that an individual that is experiencing visual stress during reading likely is experiencing a challenge with sensory input modulation, and so the presence of a color overlay reduces the attentional cognitive load required to filter visual input. This reduction via the overlay allows cognitive resources to then be reallocated to processes that support decoding and comprehension of the text. Conversely the individual selection of an overlay could be seen as motivational, leading to an intrinsic cognitive support for attention, which in turn would increase participation and engagement. Therefore, the benefit of an overlay could be seen as a behavioral response to a support for attention or state arousal which supports all other cognitive processes such as memory, problem-solving, organization, reasoning, language, etc. Either interpretation would explain the functional benefit of the overlay observed in practice and in research although further research and evidence would be required to fully support or address either proposed interpretation of the underlying mechanism.

An initial motivation for the reported work was to investigate whether cortical white matter damage in frontoparietal networks might explain visual deficits associated with mTBI. Oculomotor deficits persist in participants with TBI years after the initial injury (Fortenbaugh et al., 2021; Mani et al., 2018); he persistence of these symptoms indicates that there is likely permanent damage to the structures of the central nervous system that support eye movements. We found no association between cortical white matter integrity and convergence insufficiency or reading speed, and only a weak association with smooth pursuit movements that was not part of our planned analyses. While our study was limited in size and power, the lack of findings in controls and the weak findings in patients do not provide evidence in favor of the hypothesized cortical mechanisms. Damage to subcortical white matter structures remains a possible cause (Fortenbaugh et al., 2021; Searle and Rowe, 2016), although TBI is also associated with cranial nerve dysfunction indicating damage to these tracks could also be at play (Coello et al., 2010; Keane and Baloh, 1992).

## Conclusion

Visual dysfunction is a common consequence of traumatic brain injury. T study has highlighted the fact that reading is one of the most challenging visual behaviors people do on a daily basis. We found that individuals with mTBI were more likely to experience improved reading performance when offered a tinted overlay than control participants with no documented TBI; this improvement was predicted by high scores on the Convergence Insufficiency Symptom Survey. Our study also tested the hypothesis that decreased white matter integrity predicted high CISS scores; this hypothesis was not supported by the data, although we note that the dataset was limited in size and therefore power. The mechanisms by which changing color changes the visual system’s ability to parse text are still under investigation.

## Data Availability

The datasets presented in this study can be found in online repositories. The names of the repository/repositories and accession number(s) can be found at: https://fitbir.nih.gov/content/access-data.

## Footnotes

1
https://www.sr-research.com/eyelink-1000-plus
